# Genome-wide analysis of R2R3-MYB genes in cultivated peanut (*Arachis hypogaea* L.): Gene duplications, functional conservation, and diversification

**DOI:** 10.3389/fpls.2023.1102174

**Published:** 2023-02-14

**Authors:** Sijian Wang, Zhe Xu, Yiwen Yang, Weifang Ren, Jiahai Fang, Liyun Wan

**Affiliations:** Key Laboratory of Crop Physiology, Ecology and Genetic Breeding, Ministry of Education, Jiangxi Agricultural University, Nanchang, China

**Keywords:** cultivated peanut, R2R3-MYB transcription factors, allotetraploid, gene duplications, gene expression, functional diversity

## Abstract

The cultivated Peanut (*Arachis hypogaea* L.), an important oilseed and edible legume, are widely grown worldwide. The R2R3-MYB transcription factor, one of the largest gene families in plants, is involved in various plant developmental processes and responds to multiple stresses. In this study we identified 196 typical *R2R3-MYB* genes in the genome of cultivated peanut. Comparative phylogenetic analysis with *Arabidopsis* divided them into 48 subgroups. The motif composition and gene structure independently supported the subgroup delineation. Collinearity analysis indicated polyploidization, tandem, and segmental duplication were the main driver of the *R2R3-MYB* gene amplification in peanut. Homologous gene pairs between the two subgroups showed tissue specific biased expression. In addition, a total of 90 R2R3-MYB genes showed significant differential expression levels in response to waterlogging stress. Furthermore, we identified an SNP located in the third exon region of *AdMYB03-18 (AhMYB033)* by association analysis, and the three haplotypes of the SNP were significantly correlated with total branch number (TBN), pod length (PL) and root-shoot ratio (RS ratio), respectively, revealing the potential function of *AdMYB03-18 (AhMYB033)* in improving peanut yield. Together, these studies provide evidence for functional diversity in the *R2R3-MYB* genes and will contribute to understanding the function of *R2R3-MYB* genes in peanut.

## Introduction

MYB transcription factor is one of the most numerous families of transcription factors in plants. They are distinguished by a conserved sequence of four incomplete amino acid repeats (R), with about 52 amino acids serving as the DNA-binding amino acids. Three a-helices are formed by each repeat, and the second and third helices of each repeat create a three-dimensional (3D) HTH structure with three evenly spaced tryptophan (or hydrophobic) residues (Ogata et al., 1996). Each repeat’s third helix serves as a “recognition helix,” coming into touch with the DNA and becoming enmeshed in the main groove (Jia et al., 2004). According to the number of adjacent duplicates, MYB proteins can be classified into several types, with R2R3-MYB proteins accounting for the majority of them in plants (Dubos et al., 2010; Liu et al., 2015).

In many aspects of plant life, including primary and secondary metabolism, cell fate, developmental processes, and response to biotic and abiotic stimuli, R2R3-MYB transcription factors are crucial players (Martin and Paz-Ares, 1997; Jin and Martin, 1999; Dubos et al., 2010; Liu et al., 2015; Cao et al., 2020). The maize *C1* gene, related to the mammalian transcription factor C-MYB and is involved in the control of anthocyanin production, is the first *MYB* gene discovered in plants (Paz-Ares et al., 1987). ErMYB1 and ErMYB2 are regarded as inhibitors and activators, respectively, of the development of secondary cell walls in the eucalyptus (Goicoechea et al. 2005; Legay et al. 2007; Legay et al. 2010). ABA-mediated responses to environmental cues are mediated by the AtMYB13, AtMYB15, AtMYB33, and AtMYB101 (Reyes and Chua, 2007). AdMYB3 has been reported to be involved in anthocyanin biosynthesis and flower development in apples (Vimolmangkang et al., 2013). Members of the transcription factors that resemble MYBMIXTA are involved in starting the formation of cotton seed fiber (Bedon et al. 2014). *AhTc1*, encoding an R2R3-MYB transcription factor, play important role in regulating anthocyanin biosynthesis in peanut (Zhao et al., 2019). In rice, OsMYB30, an R2R3-MYB transcription factor, regulates the phenylalanine ammonia-lyase pathway to give brown planthopper resistance (He et al. 2020). GmMYB14 controls plant structure *via* the brassinosteroid pathway, contributing to high-density yield and drought resistance in soybean (Chen et al. 2021). MsMYB741 is involved in alfalfa resistance to aluminum stress by regulating flavonoid biosynthesis (Su et al. 2022). OsMYB60 positively regulates cuticular wax biosynthesis and this helps rice (*Oryza sativa*) plants tolerate drought stress (Jian et al., 2022).

Cultivated peanut (*Arachis hypogaea* L.), one of the most widely consumed legumes worldwide, has been used to meet the nutritional needs of developing countries globally (Toomer, 2018). It originated in South America from a heterozygous cross between two diploid ancestors and was domesticated and widely grown in the tropics and subtropics (Bertioli et al., 2016). Most of the Arachis genus is diploid (Sharma and Bhatnagar-Mathur, 2006). However, only tetraploid peanuts have been domesticated and widely grown to meet human nutritional requirements (Bertioli et al., 2016). Polyploid plants often exhibit greater environmental adaptability (Shimizu-Inatsugi et al., 2017). Recently, the contribution of polyploidization to important agronomic traits, including seed quality, fruit shape, and flowering time, has been reported for several crop species lineages such as soybean (*Glycine max*), wheat (*Triticum aestivum*), and cotton (*Gossypium hirsutum*) (Zhang et al., 2015). The large and close subgenomes of cultivated peanut genomes make genome assembly difficult (Bertioli et al., 2016). Due to advanced high-throughput sequencing technology and high-quality assembly and annotation, the sequencing of cultivated peanut was completed in 2019, the cultivated peanut (Arachis hypogaea L.) is of hybrid origin and has a polyploid genome that contains essentially complete sets of chromosomes from two ancestral species. (Bertioli et al., 2019; Chen et al., 2019; Zhuang et al., 2019). Based on high-quality whole-genome sequencing and assembly engineering, genome-wide characterization of the R2R3-MYB gene has been accomplished in various plants, such as *Arabidopsis thaliana*, rice, maize, soybean, eucalyptus, tomato, Chinese bayberry (Stracke et al., 2001; Jia et al., 2004; Chen et al., 2006; Du et al., 2012a; Soler et al., 2015; Li et al., 2016; Cao et al., 2021).

Most of the previous studies focused on the regulation mechanism of light-inducible anthocyanin (Dubos et al., 2010; Liu et al., 2015; Cao et al., 2020), but peanut is a very special and important crop. Given the fact that this crop possesses the unique characteristics of “aerial flowers and subterranean fruit,” the genes responsible for flavonoid synthesis (in peanut testa) and stress response are likely distinct from those in model plants such as *Arabidopsis* and rice. In this study, we characterized 196 R2R3-MYB transcription factors genome-wide and analyzed their phylogenetic relationships, motif composition, gene structure, chromosome distribution, gene duplication, tissue and stress response expression pattern and. Furthermore, association analysis identified a candidate gene highly correlated with total branch number (TBN), pod length (PL) and root-shoot ratio (RS ratio).Our study will contribute to the understanding of the function of the *R2R3-MYB* genes in cultivated peanut and provide candidate genes for development and stress response.

## Materials and methods

### Identification and conserved DNA-binding domain analysis of R2R3-MYBs in peanut

To identify R2R3-MYBs in peanut, the *A. hypogaea* cv. Tifrunner protein sequences were retrieved from the PeanutBase (https://peanutbase.org). The MYB DNA-binding domain (PF00249) was exploited for the identification of *R2R3-MYB* genes in the peanut genome by using the HMMER 3.3.2 program at a standard E value <1×10^–5^ (http://pfam.xfam.org/search#tabview=tab1). In total, 204 predicted gene models were found with two consecutive repeats of the MYB domain. All but five (199) were also retrieved when performing a BLASTP analysis using the previously identified genes of *A. hypogaea* against the whole *A. thaliana R2R3-MYB* gene data set (Dubos et al., 2010) with a cut-off e-value of e^-40^. Subsequently, protein sequences were evaluated for the presence of the MYB domain against the repository of the NCBI CDD (https://www.ncbi.nlm.nih.gov/Structure/cdd/wrpsb.cgi) and SMART databases (http://smart.embl-heidelberg.de/). Finally, 196 *R2R3-MYB* genes were obtained after eliminating incomplete and uncertain sequences. The predicted molecular weights and the theoretical isoelectric point (pI) were obtained by the Expasy proteomics server (https://web.expasy.org/compute_pi/). The prediction of transmembrane helices in AhR2R3-MYB proteins was analyzed by TMHMM - 2.0 (https://services.healthtech.dtu.dk/service.php?TMHMM-2.0).

The 196 R2R3-MYB protein sequences in peanuts were performed by multiple sequence alignment using ClustalX (Larkin et al., 2007). The alignment of 196 peanut R2R3-MYB domains was performed using ClustalX and DNAMAN (Lynnon Biosoft). The amino acid residue distributions of the conserved MYB domains of AhR2R3-MYBs were created using the WebLogo program with default parameters (http://weblogo.berkeley.edu/logo.cgi) (Crooks et al., 2004).

### Construction of phylogenetic tree

The protein sequences of the 126 *A.thaliana* R2R3-MYBs were downloaded from the TAIR (http://www.arabidopsis.org/). The protein sequences of R2R3-MYB proteins from *A. hypogaea* and *A.thaliana* (protein sequence information is listed in **Supplementary Table 1**) were aligned by the MAFFT with the FFT-NS-i algorithm (Katoh et al., 2019), and the multiple sequence alignments were used for phylogenetic analysis. The phylogenetic tree was constructed by the neighbor-joining method of MEGA 7.0 with 1000 bootstrap replicates based on the p-distance model and pairwise deletion for gap treatment (Kumar et al., 2016). The phylogenetic tree was retouched by FigTree (http://tree.bio.ed.ac.uk/software/figtree/). For the construction of the phylogenetic trees of R2R3-MYB proteins from *Arachis. hypogaea* the same method described above was adopted.

### Motif and gene structure analysis

Information on the intron-exon position and splicing sites for each gene model in the corresponding chromosome scaffold was downloaded from PeanutBase. Furthermore, the MEME program (https://meme-suite.org/meme/meme_5.3.3/tools/meme) was used for the identification of motifs of 196 R2R3-MYB protein sequences in peanut. The optimized parameters of MEME were employed as follows: the number of motifs that MEME find, 10; and the optimum width of each motif, 6–60 residues (Bailey et al., 2009). The gene exon-intron pattern and MEME results were also visualized by CFVisual_V2.1.5 (Chen et al., 2022).

### Chromosome localization, duplications, and evolutionary analysis of *AhR2R3-MYBs*

The information on chromosome length and *R2R3-MYB* gene locations was acquired from the PeanutBase (https://peanutbase.org) (**Supplementary Table 2**), and the figure was created by TBtools (Chen et al., 2020). The whole-genome sequences and annotation documents of *A. hypogaea* were downloaded to PeanutBase (https://peanutbase.org). Then, the One Step MCScanx program of TBtools was executed to analyze the synteny relationships of genomes. We identified gene pairs with physical distance within 100 kb, with no more than 10 genes spaced in between, and in the same subgroup as tandem repeat gene pairs according to Hanada et al. (2008). The duplication pattern of the *AhR2R3-MYB* genes was visualized by the Amazing Super Circos package of TBtools. The Ka/Ks value was completed by the Simple Ka/Ks Calculator program of TBtools. Duplication time was calculated by the following formula as described by Bertioli et al. (2016): T = Ks/2λ (λ = 8.12 × 10^−9^).

### Plant material and stress treatment

The peanut (*Arachis hypogaea* L.) cultivar ‘Changhua18’, a germplasm resource preserved in our laboratory, was planted in a pot with a 1:1 mixture of nutrient soil and vermiculite, 450 mm long, 335 mm wide, and 170 mm high. When the plants were grown for about 8 weeks, the treatment group was subjected to waterlogging treatment according to Zeng et al. (2021), while the control group was kept under normal growth conditions. The samples (three seedlings per repeat) were collected at 0 h, 6 h, 24 h, 3 days, and 5 days after treatment, respectively. Subsequently, the samples were rapidly frozen using liquid nitrogen and stored at -80°C for RNAseq.

### RNA-seq expression analysis

The raw read counts in *Arachis hypogaea* RNAseq samples were downloaded from PeanutBase (https://peanutbase.org). The data were obtained from 22 tissues at different developmental stages in peanut with three biological repeats (Clevenger et al., 2016) with all raw data deposited as BioSamples SAMN03944933–SAMN03944990. HTSeq was used to generate raw reads that were uniquely mapped on the *Arachis hypogaea* genome. StringTie and Ballgown were used for the FPKM calculation (Pertea et al., 2016). The transcript profiles for *AhR2R3-MYB* genes were displayed in TBtools (Chen et al., 2020).

These cDNA libraries generated from the samples were sequenced by Metware Biotechnology Ltd. on the Illumina sequencing platform. (Wuhan, China). Download the reference genome and its annotation files from NCBI (https://ftp.ncbi.nlm.nih.gov/genomes/), use HISAT v2.1.0 to construct the index and compare clean reads to the reference genome. The featureCounts v1.6.2/StringTie v1.3.4d was used to calculate the gene alignment and FPKM. Gene expression patterns were also charted by TBtools. Quantitative RT-PCR (qRT-PCR) was performed to verify the transcriptome data. RNAprep Pure Plant Plus Kit (Tiangen Biotech, Co., Beijing, China) was utilized to extract RNA from control and treated peanut samples. The PrimeScrit^TM^ RT Kit with gDNA eraser user manual was used to prepare the cDNA (perfect real-time, Takara Biomedical Technology, Ltd., Beijing, China). The primers were designed by TBtools and were shown in **Supplementary Table 3**. Subsequently, qRT-PCR was performed using the ABI 7500 qRT-PCR detection system (ABI, United States) with SYBR Green Kit (Tiangen, Beijing, China). The ABI 7500 real-time PCR program was 95°C for 15 min, followed by 40 cycles of 95° C for 10 s, and 60°C for 30 s in a 20 µl volume. Three technical repeats of qRT-PCR were carried out, and the relative expression level was determined using 2^-△△Ct^ technique.The results of differential expression analysis between homeolog pairs of A and B subgenomes across tissues and pod developmental stages in *Arachis hypogaea* were downloaded from PeanutBase (https://peanutbase.org) (Bertioli et al., 2019). Differential gene expression analysis was performed using the DESeq2 package (v1.14.1) with log_2_ fold change >= 1 and Benjamini-Hochberg adjusted P-value < 0.05 as the statistical cutoff for differentially expressed genes. Charts generated by GraphPad Prism 9.

### Association analysis of the peanut *R2R3-MYBs* with TBN, PL and RS ratio

Total branch number, length of root and shoot, pod size and RS phenotypes were assessed using a randomized complete block design and replicated in five environments. SNPs of the *R2R3-MYBs* were obtained from transcriptome data set of a peanut germplasm population with 146 accessions (alleles in each polymorphism with minor allele frequency >0.05). Association analysis was performed with TASSEL 3.0 using an MLM Q + K model. Figures were generated by GraphPad Prism 9.

## Results

### Identification and conserved DBD analysis of R2R3-MYB genes in peanut

A total of 196 *R2R3-MYB* genes were obtained after genome-wide screening and exclusion in *A. hypogaea* cv. Tifrunner. The 196 genes were named *AhMYB001*-*AhMYB196* according to their physical location on chromosome. The mRNA length of the *AhR2R3-MYBs* ranged from 753 to 3712 bps (**Supplementary Table 4**); the proteins length ranged from 192 to 916 amino acids, with predicted molecular weights from 21.95 to 103.72 kDa; the theoretical isoelectric point (pI) ranged from 4.60 to 10.32. Furthermore, none of the proteins were predicted to contain transmembrane domains (**Supplementary Table 4**).

To investigate the MYB conserved domains of R2R3-MYB transcription factors in peanut, we performed WebLogo and multiple alignment (ClustalX) analysis using amino acid sequences of the R2R3 repeats (**Supplementary Figure 1**). The results showed that the R2 and R3 repeat consisted of two repeats of approximately 51 amino acid residues, and all of them contained highly conserved tryptophan residues (Trp, W) (**Supplementary Figure 2**). The R2 repeat contains three highly conserved W residues at positions 5, 26, and 47, while only two highly conserved W residues were uncovered at positions 24 and 43 in the R3 repeat, and the W residue at position 5 was generally replaced by phenylalanine or isoleucine (Phe, F or Ile, I). Tryptophan residues formed a hydrophobic core maintaining the stability of the helix-turn-helix (HTH) structure in the DNA binding domain, and the basic and polar amino acids adjacent to that tryptophan were thought to be directly involved in DNA binding (Saikumar et al., 1990). Several highly conserved polar amino acid residues were also found around the third W residue in each repeat, for example, asparagine (Asn, N), arginine (Arg, R), and lysine (Lys, K) around the third tryptophan in the R2 repeat, and K and N near the third W residue in the R3 repeat, which might be directly involved in binding to DNA. These polar amino acids, which are conserved around tryptophan, were also highly conserved in different species, such as *Arabidopsis* and tomato (Stracke et al., 2001; Li et al., 2016). Overall, most of the conserved amino acid residues were mainly distributed between the second and third conserved tryptophan residues, with the first tryptophan residue being relatively less conserved. Therefore, those conserved residues probably maintain the function of the DNA binding domain together with the conserved tryptophan.

### Phylogenetic analysis of AhR2R3-MYBs in cultivated peanut and *Arabidopsis*

**Figure 1:**
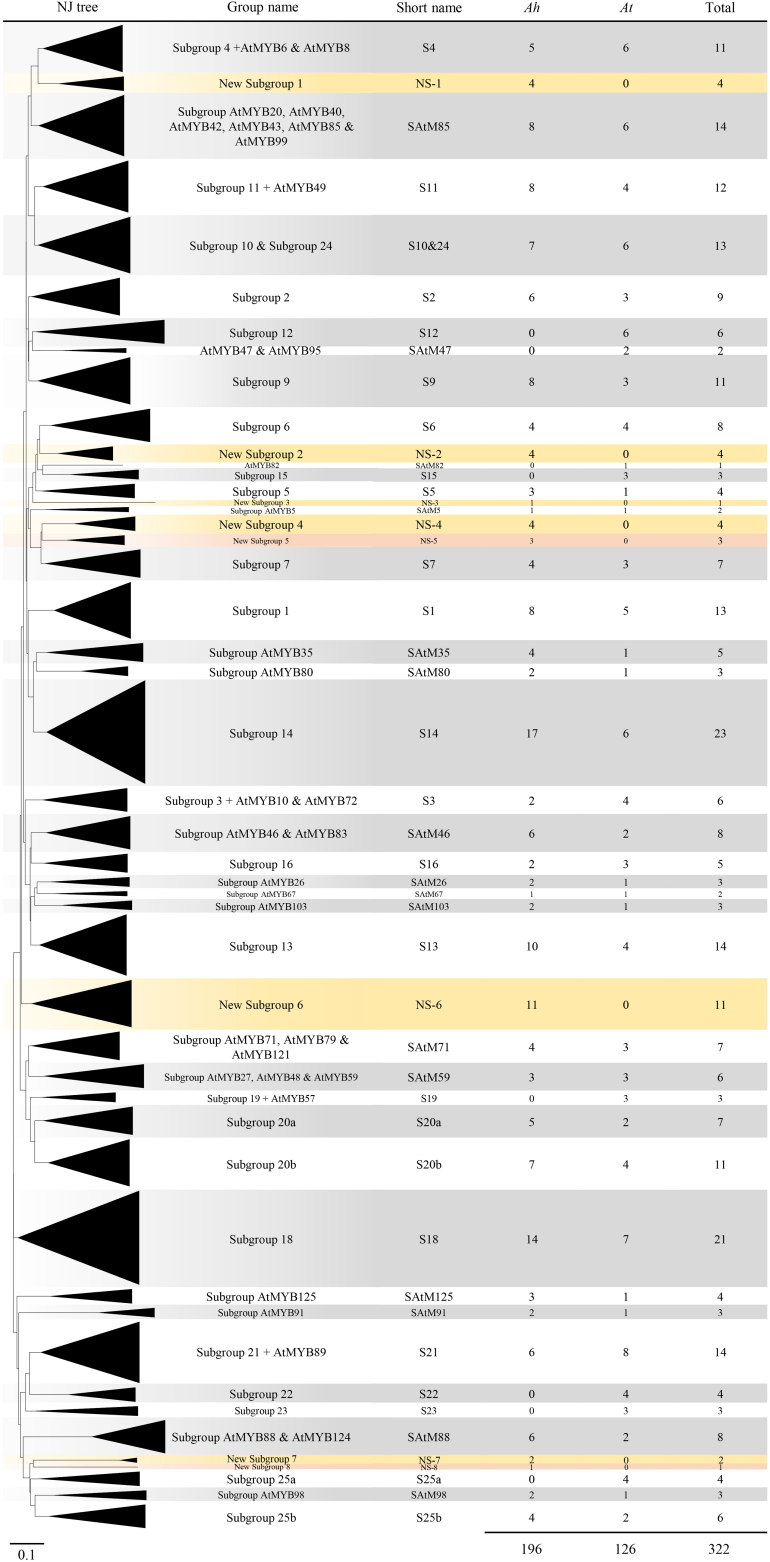
Neighbor-joining phylogenetic tree of R2R3-MYB proteins from peanut (*Arachis hypogaea *L.) and *Arabidopsis*. Each triangle represented an R2R3-MYB subgroup, defined based on the topology of the tree and the bootstrap values. Subgroup names were included next to each clade together with a short name to simplify the nomenclature. The number of genes of each species for each subgroup was also included. The eight new subgroups in peanut were marked in yellow or coral, while the other subgroups were in gray and white.

To investigate the evolutionary relationship between the *R2R3-MYB* genes in peanuts and *Arabidopsis*, the 196 predicted AhR2R3-MYB proteins were subjected to multiple sequence alignment along with 126 *Arabidopsis* R2R3-MYB proteins, and their evolutionary relationships were inferred by constructing a neighbor-joining phylogenetic tree. The 322 R2R3-MYB proteins were classified into 48 subgroups based on the topology and bootstrap value of the phylogenetic tree (**Figure 1** and **Supplementary Figure 3**). Since the *R2R3-MYB* genes have been intensively studied in *A. thaliana* and most subgroups contained at least one *AtR2R3-MYB* gene, we named these subgroups according to the nomenclature of (Kranz et al., 1998) revised by Stracke et al. (2001) and Dubos et al. (2010). When a subgroup name was not yet determined in *A. thaliana*, we named the subgroup after the member of *A. thaliana* with the most distinct functional characteristics. In general, the phylogenetic characteristics of *A. thaliana* described in this paper were generally consistent with those described previously (Stracke et al., 2001; Dubos et al., 2010). The only exception was the *A. thaliana* genes of the subgroups 20 and 25, were split in two respectively (S20a and S20b; S25a and S25b), and the genes from 10 and 24 were merged (S10&24). As shown in **Figure 1**, 40 subgroups contained at least one gene from peanut, and the other eight subgroups contained genes only from peanut, and they were named as new subgroups 1-8 in this study. The distribution of *AhR2R3-MYBs* in the 40 subgroups was biased, varying from one (NS-8) to 17 members (S14). Notably, the number of *R2R3-MYB* genes in almost all subgroups was unbiased in both subgenomes, revealing a close association between two subgenomes of cultivated peanut as described by (Bertioli et al., 2016; Bertioli et al., 2019). The topology of the neighbor-joining tree for *AhR2R3-MYB* genes was in good agreement with the subgroup described above (**Supplementary Figure 4**).

### Motif composition and gene structure of the *AhR2R3-MYB* genes

**Figure 2:**
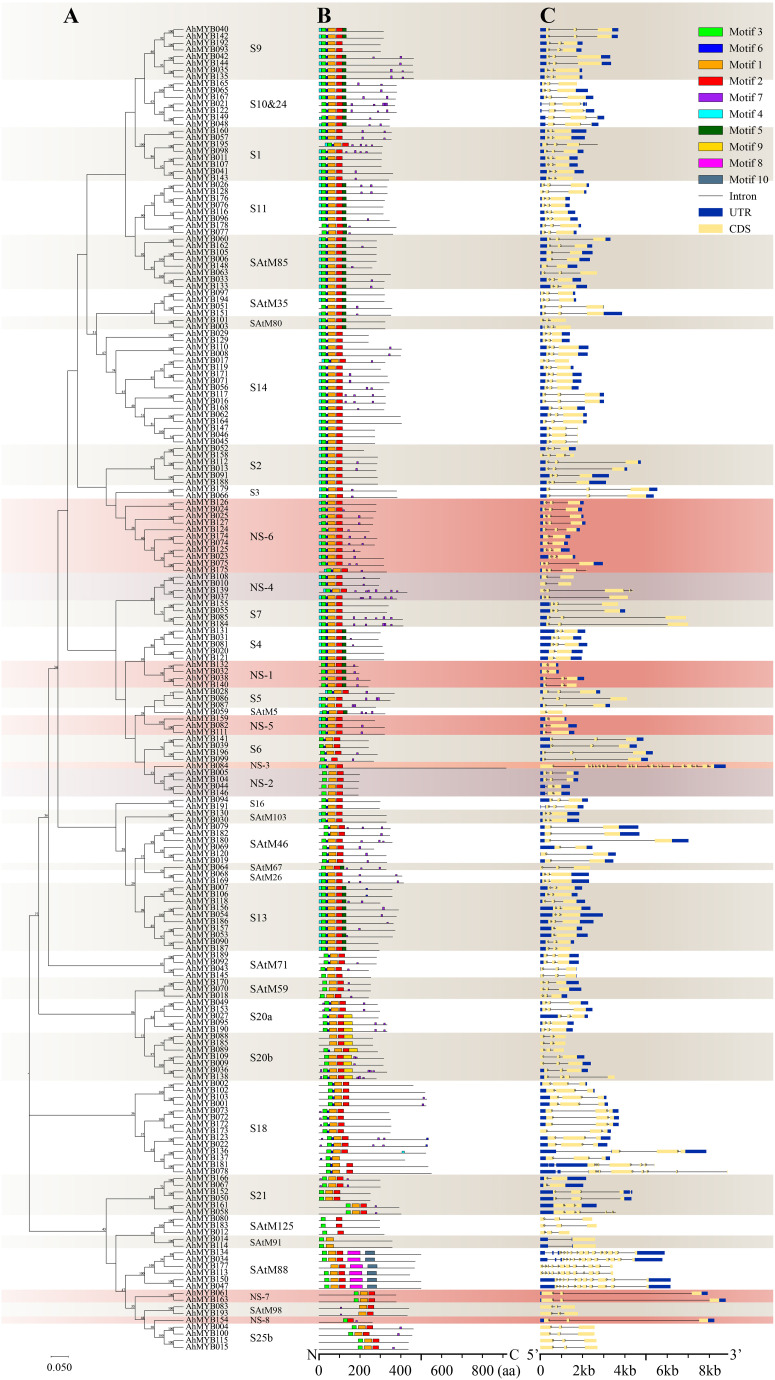
Phylogenetic relationships, conserved motifs, and gene structure analysis of peanut R2R3-MYBs. **(A)** The neighbor-joining phylogenetic tree was constructed by aligning the full-length amino acid sequences of 196 R2R3-MYBs in peanut. Coral and purple colors mark eight new subgroups, while beige and white indicate other subgroups. **(B)** The ten conserved motifs were shown in different colors and their specific sequence information was provided in **Supplementary Figure S5**. **(C)** The yellow box, blue box, and black line in the gene structure diagram represented CDS, UTR, and introns, respectively.

To investigate the relationship between subgroup classification and function of the peanut R2R3-MYBs, 10 conserved motifs were identified in the AhR2R3-MYBs through MEME program search (**Supplementary Figure 5**). The DNA binding domain of AhR2R3-MYB was represented by motifs 3, 6, 1, 2. Motifs 1 and 2 contained the amino acid sequence of the third helix forming the MYB domain, which is involved in the recognition and binding of cis-acting elements (Ogata et al., 1996; Jia et al., 2004). Motifs 5 and 8 were only presented in SAtM88, while motif 9 only presented S20a and S20b, suggesting potential specific functions of these subgroups (**Figures 2A, B**). In general, most of the motif compositions of members in the same subgroup were similar at N-terminal, but differ at C-terminal, and the motif compositions of the members in different subgroups were not identical.

Exon and intron structure analysis showed that all *AhR2R3-MYB* genes possessed 1 to 21 exons (**Figure 2C**). In general, the size of introns was variable, but the locus and phase of introns were relatively conservative among subgroups (Jiang et al., 2004). Most of the *R2R3-MYB* genes (77%) contained two conserved introns, 14% contained one conserved intron, and the rest showed a different number. Genes in the same subgroup have similar gene structures and highly conserved in intron phasing (**Figures 2A, C**). The multiple introns of a gene provide the opportunity to selectively splice and provide variant proteins that may play different roles in biological processes (Min et al., 2015). Most members of the subgroups are intron-poor (Contain three or fewer) or intron-less genes. However, we found that genes in subgroups NS-3, S18, and SAtM88 possessed an abundant number of introns, and multiple transcripts were present in all three subgroups except for *AhMYB177*.

### Chromosome localization, duplication, and evolution of the R2R3-MYB genes

**Figure 3:**
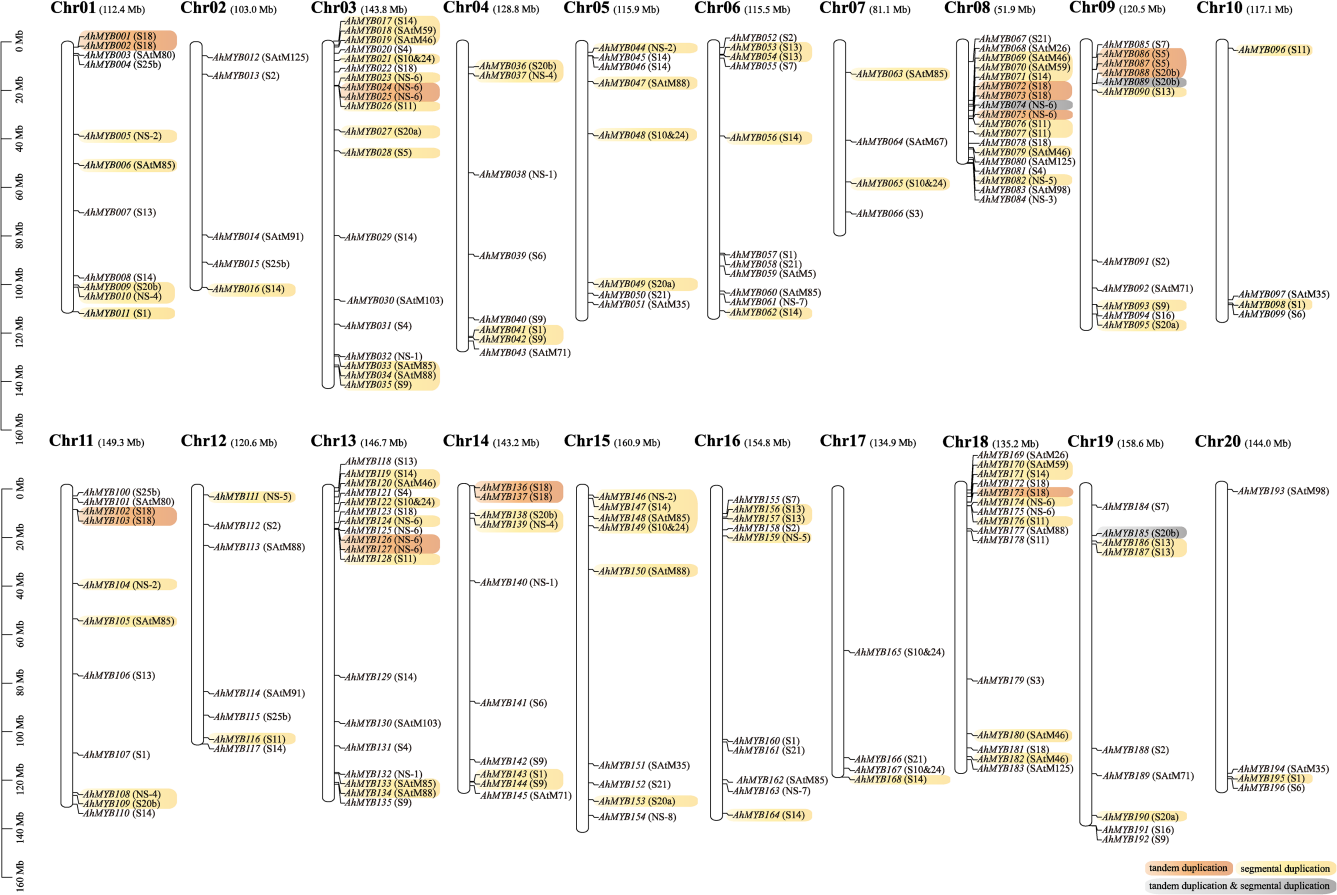
Distribution of 196 *R2R3-MYB* genes on 20 chromosomes. The name and length of the chromosome are displayed at the top of each chromosome. Yellow is for genes produced by segment duplication, orange is for genes from tandem duplication, and gray indicated genes that have experienced both types of duplication events. Subgroups were annotated to the right of the gene.

Chromosomal localization showed that the A and B subgenomes contained 99 and 97 R2R3-MYB genes, respectively (**Figure 3**). This suggested that the distributions of *R2R3-MYB* genes between the two subgenomes were almost not biased. In addition, we found that the distribution of genes on the corresponding chromosomes was similar between A and B subgenomes, except for Chr07 and Chr17, Chr08 and Chr18. This was probably the result of the complex rearrangement event on chromosomes 7, and 8 of two diploid wild ancestors, and subsequent retention of this rearrangement after polyploidization (Bertioli et al., 2016; Bertioli et al., 2019). The *R2R3-MYB* genes were unevenly distributed among the 20 chromosomes. Chr03 (19), Chr08 (18), Chr13 (18), and Chr18 (15) contained a larger number of *R2R3-MYB* genes. Chr02 (5), Chr07 (4), Chr10 (4), Chr17 (4), and Chr20 (4) possessed fewer *R2R3-MYB* genes. The density of *R2R3-MYB* genes on Chr08 was significantly higher than that on the other 19 chromosomes.

**Figure 4:**
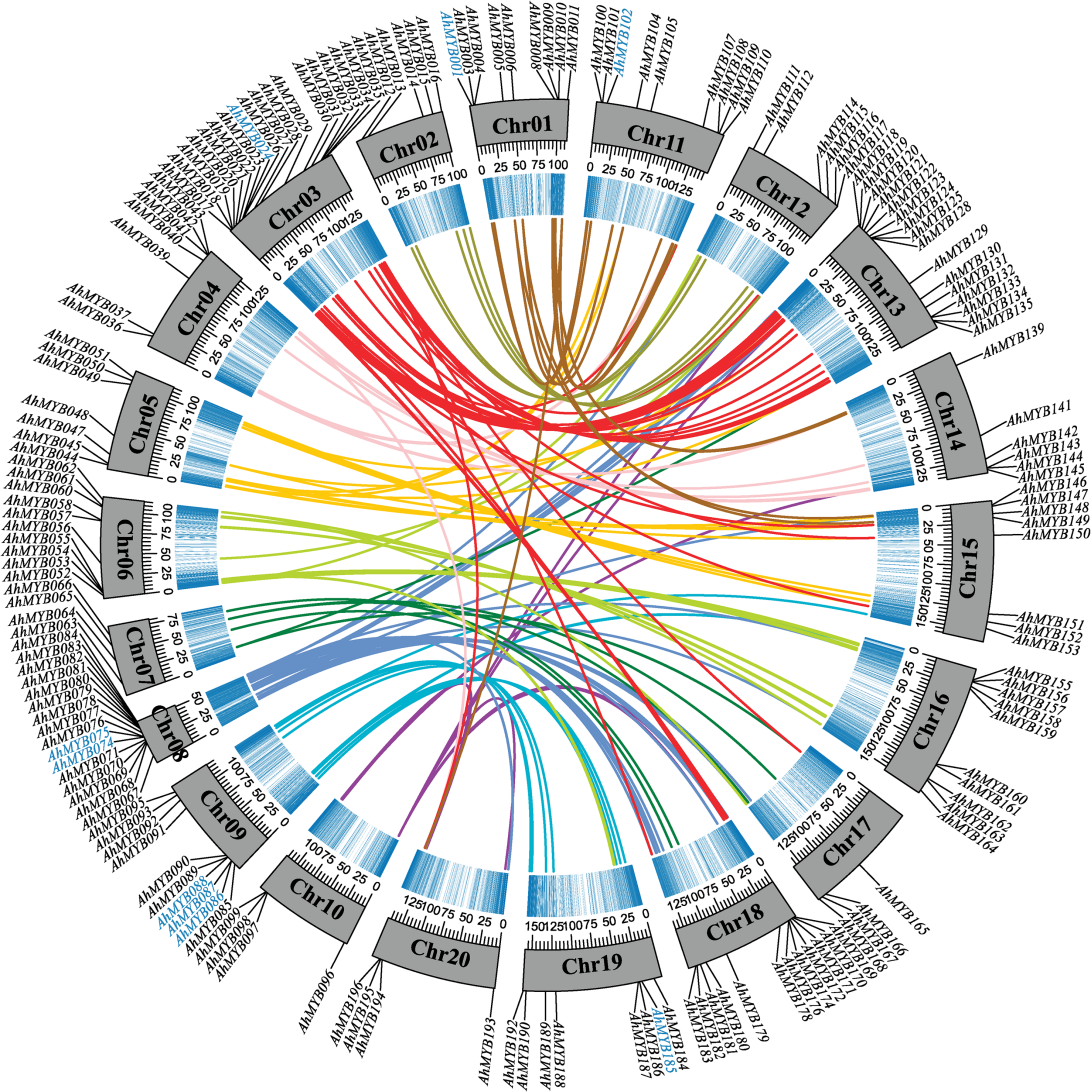
Circos diagram depicting the relationships of the chromosomes of *A. hypogaea.* Different colored connecting wires were used for different chromosome pairs. The blue color represented the density of genes. The scale for the gray bars was in megabases. Tandem duplication genes were highlighted in blue.

The *R2R3-MYB* genes were far more abundant in *A. hypogaea* than in lower terrestrial plants, suggesting that a large-scale gene duplication event occurred during the evolution of the plants (Du et al., 2015). To explore the mechanism of *R2R3-MYB* gene expansion in the cultivated peanut, we further analyzed the syntenic relationships between the peanut *R2R3-MYB* genes. Based on synteny analyses, 45 genes out of the 33 syntenic pairs in the A subgenome underwent segmental duplication events, while 39 genes out of the 24 syntenic pairs in the B subgenome were undergoing segmental duplication events (**Figure 3** and **Supplementary Table 5**). 12 and eight genes in the A and B subgenomes, respectively, experienced tandem duplication episodes. In addition, significant numbers of orthologous genes were found between the A, and B subgenomes (**Figure 4** and **Supplementary Table 5**). Based on synteny analyses, 173 of the 196 *AhR2R3-MYBs* had syntenic relationships between the two subgenomes. We found that some subgroups were expanded mainly by segment duplication, such as subgroups S11, NS-2, NS-4, SAtM85, S20a, S20b, S13, S1, S10&24, SAtM88, SAtM46, SAtM59, and NS-5, while tandem duplication occurred mainly in subgroups S5, NS-6, S20b, and S18 (**Figure 3**).

To further understand the evolution of R2R3-MYB duplication in peanut, we analyzed the rate of synonymous (Ks) and nonsynonymous substitutions (Ka) in gene duplication pairs (**Supplementary Table 5**). The Ks values for direct homologous gene pairs between A and B subgenomes range from 0 to 2.47. The frequency distribution peaks at Ks = 0.03, indicating a massive duplication event of *R2R3-MYB* genes 1.85 million years ago (Mya). Segment duplication and tandem duplication may occur in 147.75-35.91 and 163.87-2.20 Mya, respectively. Ka/Ks ratios for paralogous and orthologous ranged from 0-2.10 with an average of 0.27, whereas the ratios for tandem duplication ranged from 0.13-1.02 with an average of 0.36. The Ka/Ks analysis showed that the orthologous gene pair *AhMYB052*-*AhMYB158* was neutrally selected and the tandem duplication pair *AhMYB173*-*Arahy.QI53CA* and the orthologous gene pairs *AhMYB010*-*AhMYB108*, *Arahy.M1LASL*-*AhMYB118*, and *AhMYB031*-*AhMYB131* were subjected to positive selection, and all other synteny and tandem duplicated genes were subjected to purifying selection.

### Tissue expression profiles of the *R2R3-MYBs*

**Figure 5:**
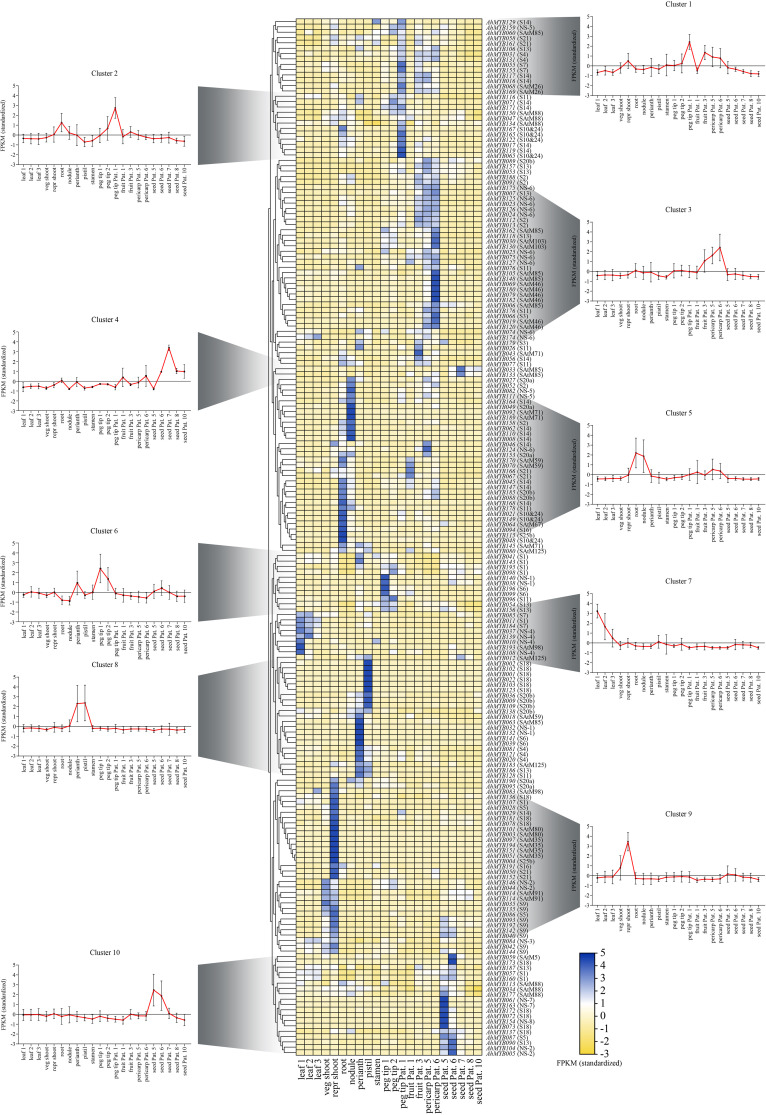
Heat map of the RNAseq transcript abundance pattern of the 194 R2R3-MYB genes in 22 different tissues in 10 expression clusters. For each gene, its name and the subgroup it belongs to were displayed on the right side of the heatmap. The expression pattern was generated based on the fragments per kilobase of exon per million fragments (FPKM) and analyzed by heatmap hierarchical clustering. The color scale (representing −2 to 5) was shown. The meanings of the abbreviations of the 22 tissues were as follows: seedling leaf 10 days post-emergence (leaf 1), main stem leaf (leaf 2), lateral stem leaf (leaf 3), vegetative shoot tip from the main stem (veg shoot), reproductive shoot tip from first lateral (repr shoot), 10-day roots (root), 25-day nodules (nodule), perianth, stamen, pistil, aerial gynophore tip (peg tip 1), subterranean peg tip (peg tip 2), Pattee 1 stalk (peg tip Pat. 1), Pattee 1 pod (fruit Pat. 1), Pattee 3 pod (fruit Pat. 3), Pattee 5 pericarp (pericarp Pat. 5), Pattee 6 pericarp (pericarp Pat. 6), Pat - tee 5 seed (seed Pat. 5), Pattee 6 seed (seed Pat. 6), Pattee 7 seed (seed Pat. 7), Pattee 8 seed (seed Pat. 8), and Pattee 10 seed (seed Pat. 10).

To further study the expression pattern of the *R2R3-MYB* genes in different tissues and explore its function in peanut growth and development, the tissue expression profiles of the *R2R3-MYB* genes were analyzed by using the transcriptome data of 22 peanut tissues (*AhMYB015* and *AhMYB100* were not detectable in the dataset) (**Figure 5** and **Supplementary Table 6**). The peanut R2R3-MYBs can be clustered into 10 groups according to their tissue expression pattern (Cluster 1-10). Most genes from same phylogenetic subgroup showed similar tissue expression patterns and were clustered into the same cluster, such as *AhMYB023*, *AhMYB024*, *AhMYB074, AhMYB125*, *AhMYB126*, *AhMYB174*, and *AhMYB175* in NS-6, which highly expressed in fruit and pericarp, were cluster in cluster 3 (**Figure 5**). All the members of subgroup SAtM35, for example, were clustered in cluster 9 (**Figure 5**). However, a certain proportion of the *AhMYBs* from the same phylogenetic subgroup showed differential tissue expression, such as *AhMYB028* and *AhMYB087* from S5, *AhMYB028* was highly expressed in the reproductive shoot, while *AhMYB087* was mostly expressed in seed (**Figure 5**), this might be because genes belonging to the same phylogenetic subgroup exercise similar functions, but in different tissues responses to different developmental processes or different environmental stimuli ( (Dubos et al., 2010).

Interestingly, members of the eight peanut specific subgroups (NS-1 to 8) were distributed in all clusters except 2 and 4.The two NS-1 members (*AhMYB032* and *AhMYB132*) were strong expressed in perianth; the two collinear gene pairs (*AhMYB005* and *AhMYB104*, *AhMYB044* and *AhMYB146*) from NS-2 subgroup were highly expressed in seed and shoot, respectively. *AhMYB084* (NS-3) had significant expression both in leaf and shoot; the three members in NS-4 (*AhMYB010*, *AhMYB037* and *AhMYB139*) expressed mainly in leaf; *AhMYB082* and *AhMYB111 in* NS-5 showed higher expression level in nodule, but *AhMYB150* was enriched in peg tip; most of the members in NS-6 were clustered in cluster 3 and were highly expressed in pericarp; members in NS-7 (*AhMYB061* and *AhMYB163*) and NS-8 exhibited strong expression in seed. These subgroups contained totally 30 genes, 21 of which were expressed at high levels in reproductive organs, especially the 10 genes in NS-6 that had high transcript abundance in the early pod development stage after peg tip entry, suggesting an important role of these genes in peanut reproductive development.

### Comparison of R2R3-MYB gene expression in subgenomes

**Figure 6:**
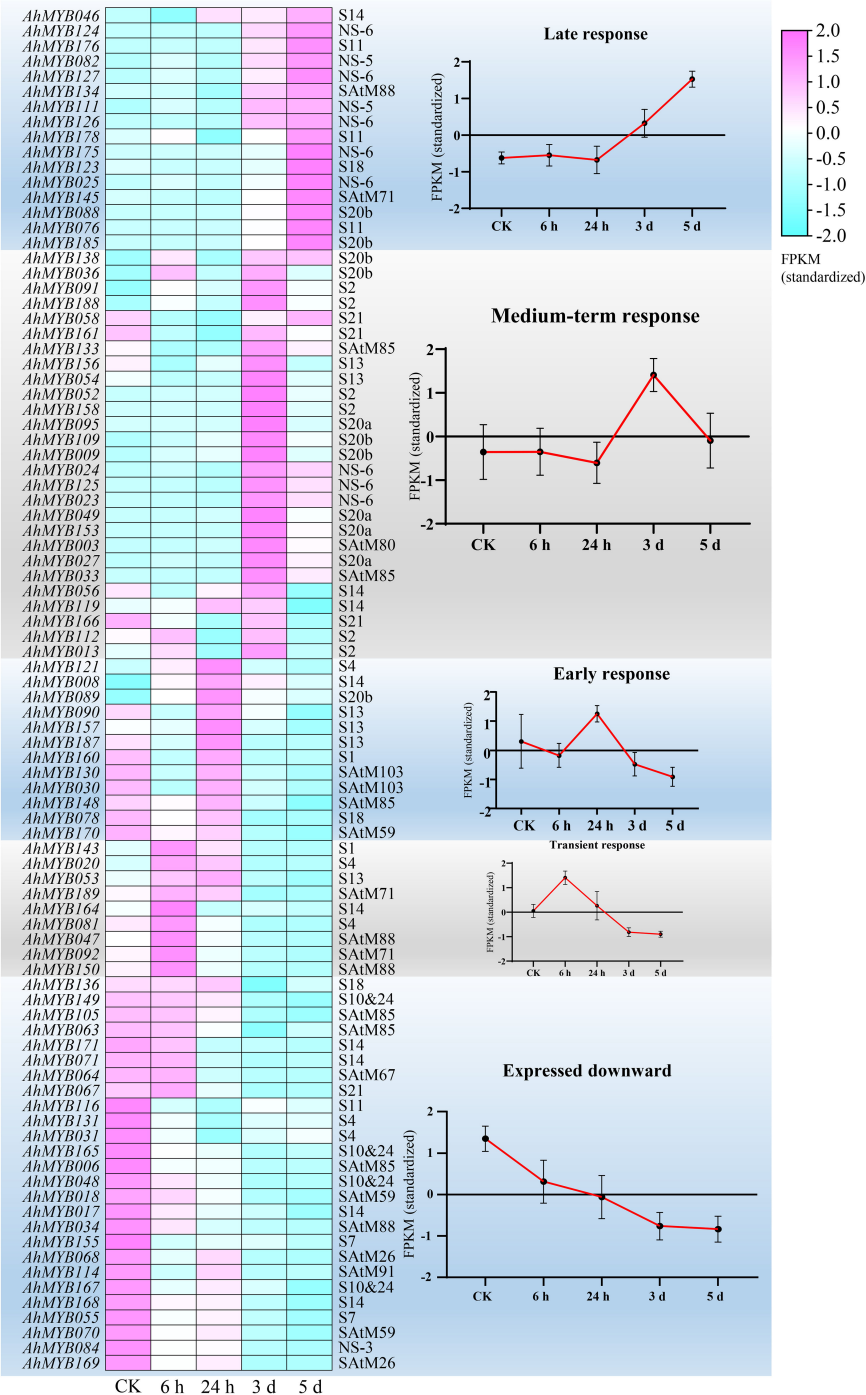
RNAseq transcript abundance patterns of 90 R2R3-MYB genes in root and stem tissues in five expression groups during water logging stress are shown as a heat map. The left and right sides of the heatmap, respectively, showed the names of each gene and the subgroup to which it belonged. Heatmap hierarchical clustering was used to construct the expression pattern based on the fragments per kilobase of exon per million fragments (FPKM). The color scale, which ranged from -2.0 to 2.0, was displayed. The labels at the bottom of the heat map indicate, from left to right, the control group, 6 h, 24 h, 3 days, and 5 days after water logging treatment.

The expression of homeologous gene pairs from A and B subgenomes was examined in various tissues and developmental phases (**Supplementary Table 7** references from (Bertioli et al., 2019)). The total number of homeologous gene pairs with expression biased towards the A subgenome was similar to the previously reported it did not differ significantly from the number with expression biased towards the B subgenome (P = 0.16, binomial test; n = 47 and 42 for A and B, respectively) (**Figure 6**) (Chalhoub et al., 2014; Zhang et al., 2015). In nine tissues (lateral leaf, seeding leaf, vegetative shoot tip, reproductive shoot tip, perianth, gynoecium, pattee 6 seed, pattee 7 seed, and pattee 8 seed), of the homologous pairs, there were more A subgenome-highly expressed genes rather than B subgenome, whereas the other 10 tissues exhibited the reverse pattern. These differences were more significant in the four reproductive tissues (P < 0.05, binomial test). 14 homologous gene pairs had biased expression in just one tissue whereas 18 homologous gene pairs exhibited the same bias in several tissues (**Supplementary Table 8**). Interestingly, we identified four homologous gene pairs (*AhMYB006* and *AhMYB105*, *AhMYB011* and *AhMYB107*, *AhMYB053* and *AhMYB157*, *AhMYB074* and *AhMYB174*) showed opposite biased expression in specific tissues, such as homolog pair *AhMYB074* and *AhMYB174*, *AhMYB174* owned a higher expression level in lateral leaf {log2FoldChange (B.vs.A homeolog pair comparison):1.906081616}, while *AhMYB074 had a stronger expression level in perianth* {log2FoldChange (B.vs.A homeolog pair comparison): -2.204691792}.

### Expression pattern of *R2R3-MYB* genes in peanut under waterlogging treatment

**Figure 7:**
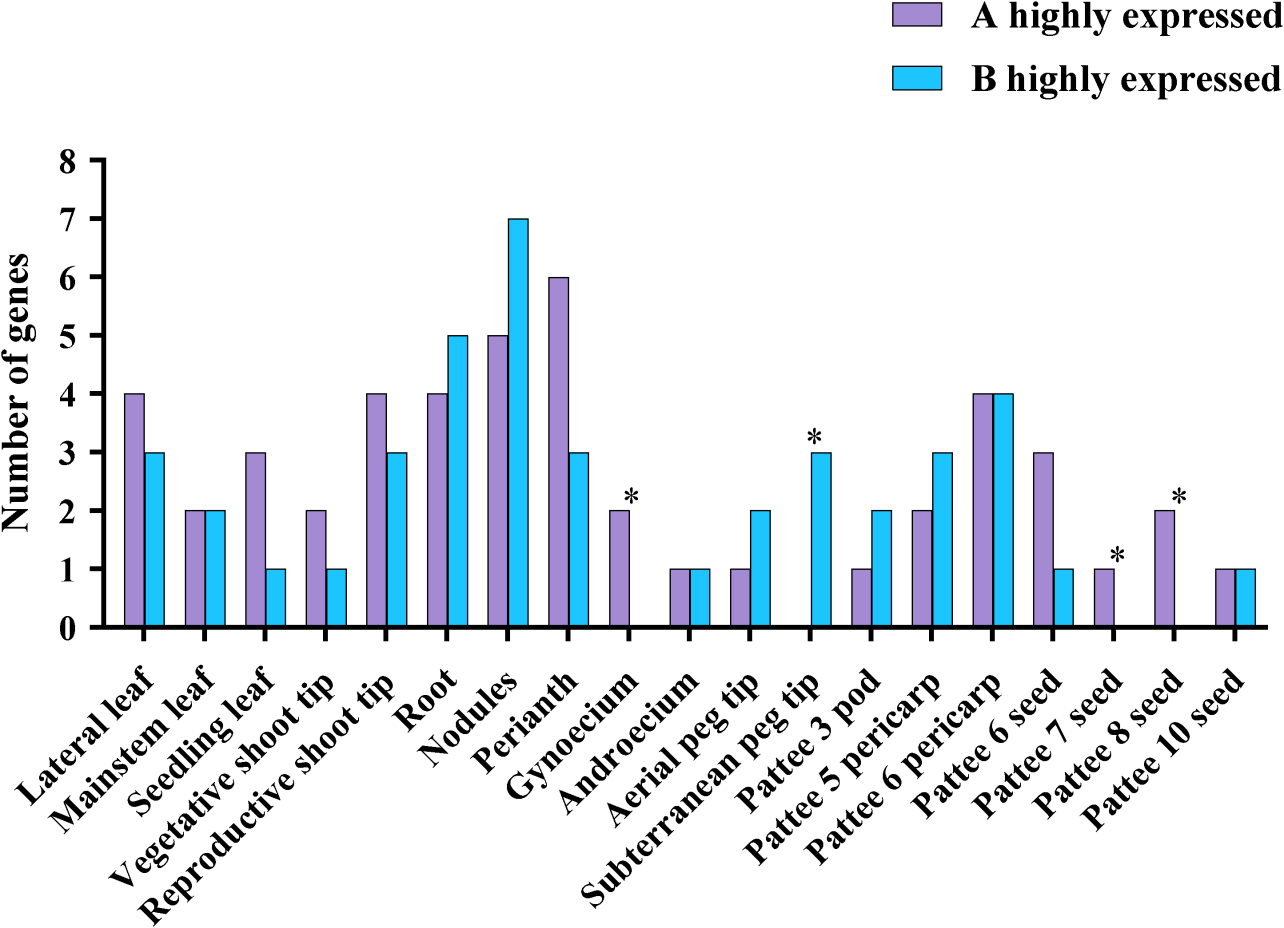
Differential expression of R2R3-MYB homologous genes in *A. hypogaea* cv. Tifrunner. To look for variations in the levels of gene expression in 19 distinct organs, homeologs were compared. Each subgenome was represented by the number of homeologous genes that were more strongly expressed (log2 fold change ≥ 1, Benjamini-Hochberg adjusted P < 0.05; Wald test) in each subgenome is represented. P-value correspond to binomial test with the odds of A genes being more highly expressed at 0.5 probability. *P < 0.05, others : not significant.

We examined the transcript abundance of the R2R3-MYB gene in peanut seedlings after water logging treatment to further investigate the function of the genes in water logging response. 90 genes from 24 subgroups (NS-6, S10&24, S11, S13, S14, S2, S20a, S20b, S21, SAtM85, SAtM88, etc.) were found response to waterlogging. According to the chronological order of the respond genes, they were divided into five categories (**Figure 7**). 26 genes showed a strong tendency to be down-regulated following the water logging treatment. Nine genes in the transitional response group showed strong elevated after 6 hours of treatment. Three genes (*AhMYB121*, *AhMYB008*, and *AhMYB089*) were considerably up-regulated among the 12 early responded genes, whereas the others showed a tendency of down- and subsequently up-regulation. At the time point of 3 and 5 days after treatment, a total of 27 and 16 genes were up-regulated, respectively. In addition, the results of qRT-PCR showed that the expression patterns of the 10 selected genes under water logging treatment were generally consistent with the transcriptome results (**Supplementary Figure 6**) which verified the results based on transcriptome data analysis.

### Association analysis of R2R3-MYBs with pod size, total branch number and root-shoot ratio of peanut

**Figure 8:**
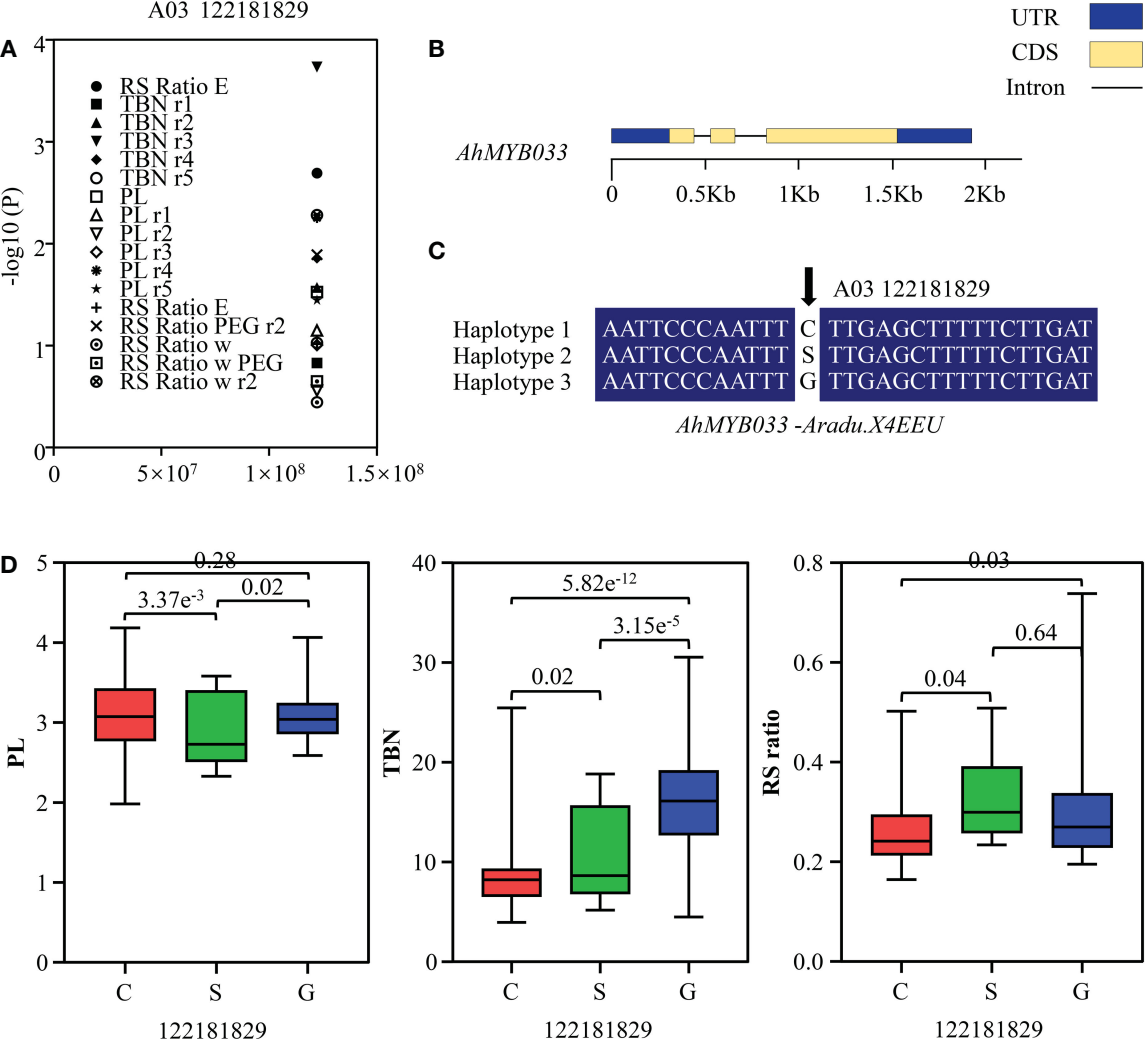
Association mapping results and the phenotypes of the polymorphic sites of peanut R2R3-MYBs associated with TBN, PL and RS ratio variation. **(A)** Association results between TBN/PL/RS ratio and the polymorphisms in *AhMYB033*. The vertical and horizontal axes of the scatter plot indicated the P value and the position of the SNP, respectively, and the phenotypes under different environmental treatments were represented by various patterns. **(B)** Gene structure of *AhMYB033*. The yellow box, blue box, and black line were CDS, UTR, and intron, respectively. **(C)** Sequences of three sites were significantly associated with TBN, PL and RS ratio variation. **(D)** Phenotypic comparison of haplotypes of the three associated sites with TBN, PL and RS ratio in five environments of the population. Three different colored boxes were used to indicate the phenotypes of the three haplotypes.

To determine the role of *R2R3-MYB* genes in peanut, we conducted candidate gene association analysis using 59 single-nucleotide polymorphisms in *Ad* and *AiR2R3-MYBs* identified from transcriptome data of 146 peanut varieties (**Supplementary Table 9**) and phenotype of total branch number (TBN), pod length (PL) and root-shoot ratio (RS ratio) variation collected in five environments (**Supplementary Table 10**). One polymorphic site [A03_122181829 (C/S/G)] was uncovered highly associated with TBN, PL and RS ratio variation in five environments (P < 0.01) (**Supplementary Table 11** and **Figure 8A**), located in the third exon region of *AdMYB03-18 (AdMYB03-18)* (**Figure 8B**). A03_122181829 mainly formed three haplotypes (A03_122181829 (C/S/G)) in the associated population (**Figure 8C**). Analysis results indicated that TBN in haplotype G was notably higher than those in haplotype C, while PL in haplotype C was higher than PL in haplotype G and S, RS ratio in haplotype S was higher than RS ratio in haplotype C and G (**Figure 8D**).

## Discussion

The R2R3-MYB genes have been identified in various species, such as *Arabidopsis*, rice, maize, soybean, eucalyptus, tomato, and Chinese bayberry (Stracke et al., 2001; Jia et al., 2004; Chen et al., 2006; Du et al., 2012a; Du et al., 2012b; Soler et al., 2015; Li et al., 2016; Cao et al., 2021). In this study, 196 *R2R3-MYB* genes were identified from cultivated peanut (*Arachis hypogaea* L.), which were further characterized by co-phylogenetic analysis with the corresponding genes of *A. thaliana*. According to the topology of the resulting tree and the bootstrap values, a total of 322 R2R3-MYBs were divided into 48 subgroups. Among these subgroups, the *Arabidopsis* genes of the subgroups S20 and S25 were split into two subgroups (S20a and S20b; S25a and S25b), and the genes from subgroups S10 and S24 were merged (S10&24). The combination of the two subgroups into one group suggested that the genes in these subgroups may have close evolutionary relationships, and there is differentiation in function. In some subgroups (S12, S15, S19, S22, S23, S25a, SAtM47, AtMYB82), only genes in *Arabidopsis* were present, indicating that after peanut differentiated from *Arabidopsis*, genes were either lost in peanut or acquired in *Arabidopsis*. Moreover, in our results, there were eight subgroups (NS-1, 2, 3, 4, 5, 6, 7, 8) specific to peanut, indicating these genes might be newly expanded genes after differentiation from *Arabidopsis*. Several other genetic characteristics must be considered when performing subfamily classifications, including the presence of highly conserved intron patterns and motif distribution within each subfamily (Du et al., 2015). In this study, exon-intron pattern and motif distribution within each subgroup were also highly conserved, which independently supports our phylogeny analysis and classification results.

As genes in the same subgroup are believed to have relatively similar roles, sequence-based homology classification is crucial for developing hypotheses about the functions of R2R3-MYB genes that have not yet been studied in model species. It is also crucial for defining putative ortholog relationships with known genes in model species to ascertain the functions of genes in nonmodal species (Jiang et al., 2004). The function of novel genes in *Arabidopsis* and other species can be inferred by protein structure and expression patterns (Dubos et al., 2010). *Arabidopsis* members in subgroup S6 were involved in the phenylpropanoid pathway, which can activate the late biosynthetic genes (LBGs) leading to both anthocyanin and PA biosynthesis (Gonzalez et al., 2008), suggesting a potential regulatory function of the peanut *R2R3-MYB* genes in S6 in the phenylpropanoid metabolic pathway. (Zhao et al., 2019) identified the *AhTc1* gene encoding an R2R3-MYB transcription factor controlling peanut purple testa color by whole-genome resequencing-based QTL-seq. In this work, *AhTc1* was given the name *AhMYB099* and belongs to subgroup S6. The identification of *AhTc1* supports the above hypothesis, suggesting that these theories help to speculate on unknown gene functions. Similarly, in *Arabidopsis*, many studies have shown that members of S18 are involved in abscisic acid-mediated responses to environmental signals and in promoting anther and pollen development (Millar and Gubler, 2005; Reyes and Chua, 2007). *AtMYB37*, *AtMYB38*, and *AtMYB84*, members of subgroup S14, partly redundantly control axillary meristem tissue development in *Arabidopsis* (Keller et al., 2006; Muller et al., 2006). Under adverse circumstances, the root growth-specific regulator *AtMYB68* (subgroup 14) has an impact on the development of the entire plant. Members of subgroup S18 were grouped in gene expression clusters 8, 9, and 10. The *AhR2R3-MYB* genes in subgroup S14 were highly expressed in the subterranean peg tips, roots, and reproductive shoot tips. Therefore, it can be inferred that these genes perform similar functions as the same subgroup of *Arabidopsis* genes.

Gene expression profiles provided important threads for the study of gene function. In the present study, we also explored the role of the *R2R3-MYB* gene in water logging stress. A total of 90 genes from 24 subgroups responded to water logging stress in roots and stems. However, some of these 90 genes were not found to be highly expressed in roots and stems in the previous expression analysis of 22 tissues. For example, *AhMYB076* and *AhMYB176* were only expressed at high levels in the pericarp, and *AhMYB071* and *AhMYB171* were expressed at high levels in the underground part of the peg tip and the early developing pods. This mi ght be because these genes perform similar functions but have different expression patterns or all respond to certain environmental stimuli (Dubos et al., 2010). In addition, the peg tip of the underground parts becomes more root-like after reaching into the soil, which seems to explain the presence of these genes in the roots or stems in response to water logging stress (Kumar et al., 2019). The important roles of R2R3-MYBs in response to abiotic stress have been reported several times before in *Arabidopsis*, jatropha, sesame and maize (Dubos et al., 2010; Thirunavukkarasu et al., 2013; Juntawong et al., 2014; Mmadi et al., 2017; Yu et al., 2020). For example, AtMYB60 in subgroup S1 is involved in ABA-mediated control of stomatal opening and closing in response to drought (Cominelli et al., 2005). AtMYB15 in subgroup S2 is involved in cold stress response (Agarwal et al., 2006). AtMYB41 in subgroup S11 probably affects dehydration response after osmotic stress (Cominelli et al., 2008; Lippold et al., 2009). Several MYBs were significantly induced in jatropha, sesame and maize (Thirunavukkarasu et al., 2013; Juntawong et al., 2014; Mmadi et al., 2017; Yu et al., 2020). However, the expression pattern of R2R3-MYB genes under water logging stress exhibited significant temporal specificity. 18 genes showed a continuous down-regulation after treatment, while the remaining genes showed an up-regulation after 6 h, 24 h, 3 days, and 5 days after treatment, respectively. This stress response with a high number of participating genes and a certain temporal pattern disclosed a complex and ordered regulatory network involving *R2R3-MYB* genes in response to water logging stress.

Whole genome duplication or polyploidization occurs frequently in angiosperms and provides a great deal of material for plant evolution (Jiao, 2018). Most of the replicated genes from WGD are eventually lost (Lynch and Conery, 2000; Conant et al., 2014), and those that are retained are often biased toward certain functional gene taxa (Panchy et al., 2016). In this research, the *R2R3-MYB* gene also had a high homology ratio (88.3 %) between the two subgenomes of tetraploid peanut (*Arachis hypogaea*). As previously reported in *Arabidopsis* and Brassica, the genes that are typically retained (and therefore enriched) are kinases, transcriptional proteins, transcription factors, and genes functioning in transcriptional regulation (Maere et al., 2005; Liu et al., 2014). High retention of the *R2R3-MYB* homolog after polyploidization in peanut reveals functional conservation of the *R2R3-MYB* gene. Under natural selection, the ploidized genes experience different fates, such as partial copy loss and loss of function (pseudogenization), partial copy gaining new function, or each exercising part of the function of the ancestral gene (Conant and Wolfe, 2008; Panchy et al., 2016). The results showed that, in general, the homologous gene pairs were not significantly biased to be expressed between the two subgenomes. However, considering only one tissue, homologous pairs showed biased expression among subgenomes in the four reproductive tissues. A total of 32 homologous gene pairs exhibited biased expression between subgenomes in the same or different tissues, suggesting that some genes in these homologous pairs may be lost in function. More homologous genes did not have significantly biased expression and they may have been sub-functionalized, each exercising part of the function of the ancestral gene. Moreover, we identified four homologous pairs exhibiting different biases in different tissues, which reveal a novel functionalization of the *R2R3-MYB* gene. In conclusion, these findings revealed the fate of the *AhR2R3-MYB* genes after undergoing polyploidization and collectively maintaining a functional dosage balance.

## Conclusion

In this study, 196 *R2R3-MYB* genes were identified in cultivated peanut genome. A phylogenetic study with *Arabidopsis* divided the 196 genes into 40 subgroups. Motif composition and gene structure analyses independently confirmed the subgroup delineation. According to the synteny analysis, polyploidization, facilitated in the expansion of the *AhR2R3-MYB* genes. Tissue expression pattern of *R2R3-MYB* genes, subgroup functional conservation, and diversification were discovered using gene expression analysis. The varied outcomes of homologous genes following polyploidization were revealed by the biased expression of homologous pairs in the two sub genomes. 90 genes exhibited a clearly time-specific expression pattern when stressed by waterlogging and AhMYB33 was identified by association analysis highly correlated with total branch number (TBN), pod length (PL) and root-shoot ratio (RS ratio). In conclusion, our research advances knowledge of the role of R2R3-MYB transcription factors in cultivated peanut, particularly in response to waterlogging stress.

## Data availability statement

The raw data generated in this study have been deposited in the NCBI repository, accession number PRJNA291488.

## Author contributions

LW conceived the idea of the paper, SW and ZX carried out all the experiments and data analyses. SW, YY, and WR prepared the figures and tables. SW wrote the manuscript, LW and JF made modifications to the article. All authors contributed to the article and approved the submitted version.

## Supplementary material

Supplementary Figure 1 Consistent sequence alignment of the R2R3-MYB domain in *Arachis hypogaea*. The alignment of 196 AhR2R3-MYB domains was performed using ClustalX and DNAMAN. The shading of the alignment represents different degrees of conservation among sequences, respectively. The sites of five highly conserved tryptophan residues (W) were indicated by pentacles.

Supplementary Figure 2 Consensus sequence and the level of conservation of R2R3-type MYB domains from peanut. The sequence logos of the R2 and R3 MYB repeats were based on multiple alignment analyses of 196 typical AhR2R3-MYB domains performed with ClustalX 2.1. The vertical axis indicated the degree of amino acid conservation, and the horizontal axis indicated the position of the amino acid on each repeat. The conserved tryptophan residues (Trp, W) in the MYB domain were marked with black asterisks. The replaced residues in the R3 repeat were shown by blue asterisks.

Supplementary Figure 3 Phylogenetic NJ tree constructed with 126 *Arabidopsis* and 196 peanut R2R3-MYB proteins. Bootstrap values were on the branch node. The subgroup labels were marked on the right side of the tree.

Supplementary Figure 4 Phylogenetic NJ tree constructed using 196 AhR2R3-MYB proteins. Bootstrap values were displayed at the branch nodes. The subgroup labels were labeled on the right side of the tree.

Supplementary Figure 5 10 MEME motif sequence logos in AhR2R3-MYBs.

Supplementary Figure 6 qRT-PCR verification of the expression of *AhR2R3-MYBs* after water logging stress.
