# Supplementary figures and images for: Genome-wide analysis of R2R3-MYB genes in cultivated peanut (Arachis hypogaea L.): Gene duplications, functional conservation, and diversification
Source: Front Plant Sci. 2023 Feb 14;14:1102174. doi: 10.3389/fpls.2023.1102174 (PMC9971814; doi:10.3389/fpls.2023.1102174)

# R2

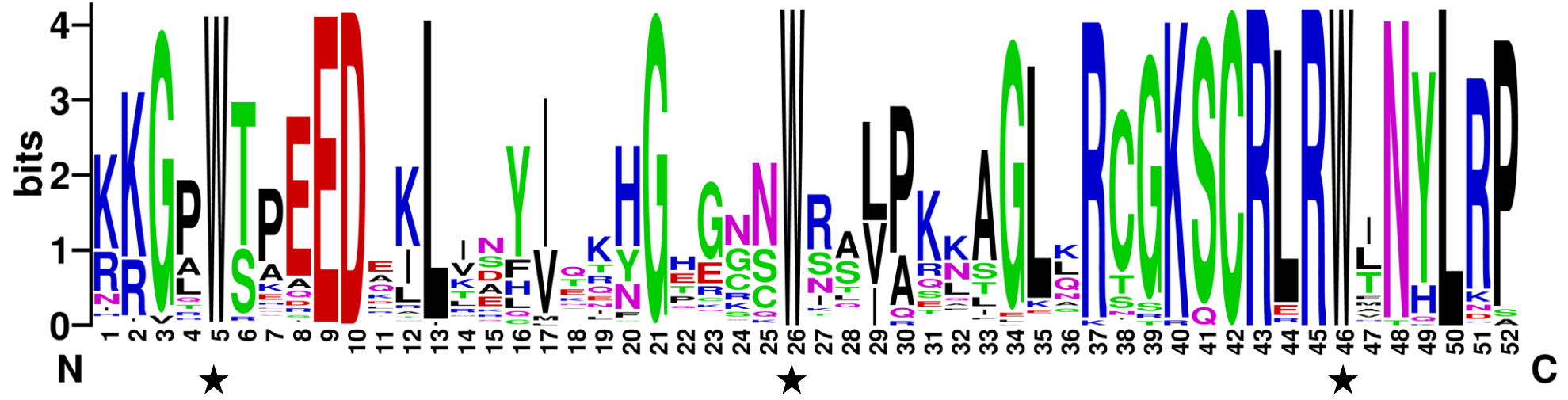

# R3

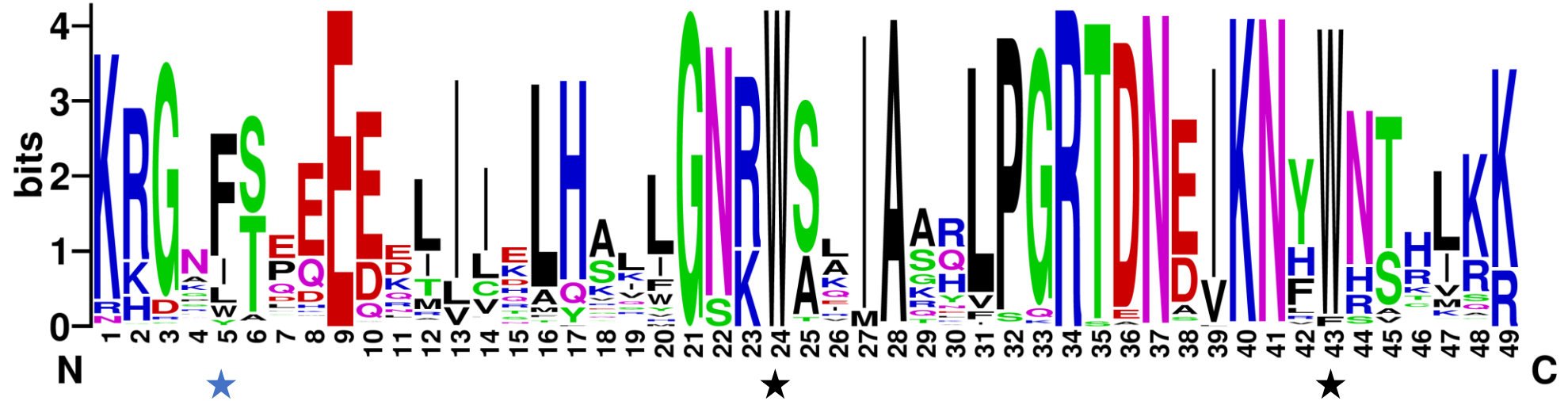

Supplement: Supplementary Figure 2 — Consensus sequence and the level of conservation of R2R3-type MYB domains from peanut. The sequence logos of the R2 and R3 MYB repeats were based on multiple alignment analyses of 196 typical AhR2R3-MYB domains performed with ClustalX 2.1. The vertical axis indicated the degree of amino acid conservation, and the horizontal axis indicated the position of the amino acid on each repeat. The conserved tryptophan residues (Trp, W) in the MYB domain were marked with black asterisks. The replaced residues in the R3 repeat were shown by blue asterisks. [file DataSheet_2.pdf]

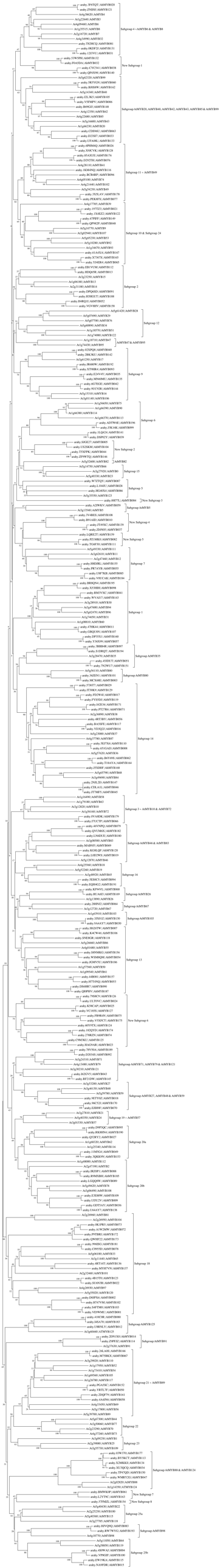

Supplement: Supplementary Figure 3 — Phylogenetic NJ tree constructed with 126 Arabidopsis and 196 peanut R2R3-MYB proteins. Bootstrap values were on the branch node. The subgroup labels were marked on the right side of the tree. [file DataSheet_3.pdf]

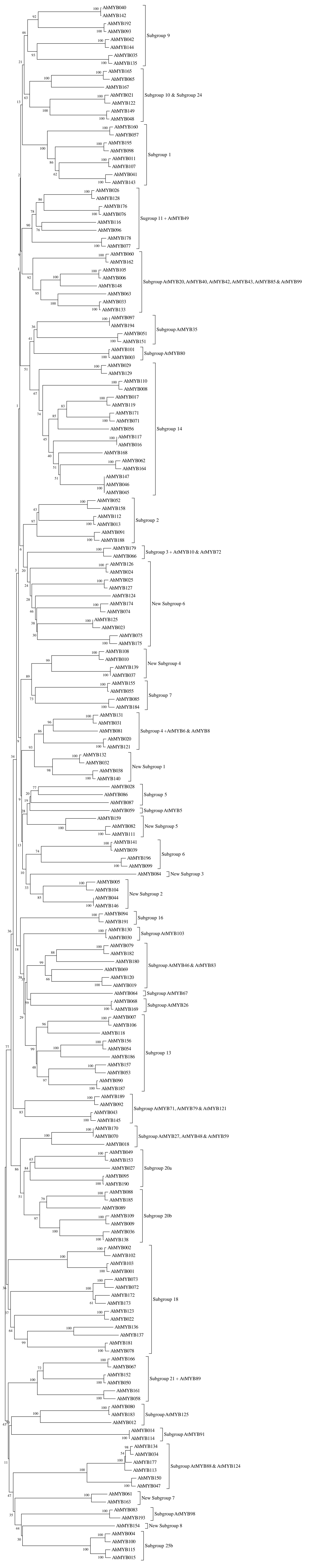

Supplement: Supplementary Figure 4 — Phylogenetic NJ tree constructed using 196 AhR2R3-MYB proteins. Bootstrap values were displayed at the branch nodes. The subgroup labels were labeled on the right side of the tree. [file DataSheet_4.pdf]

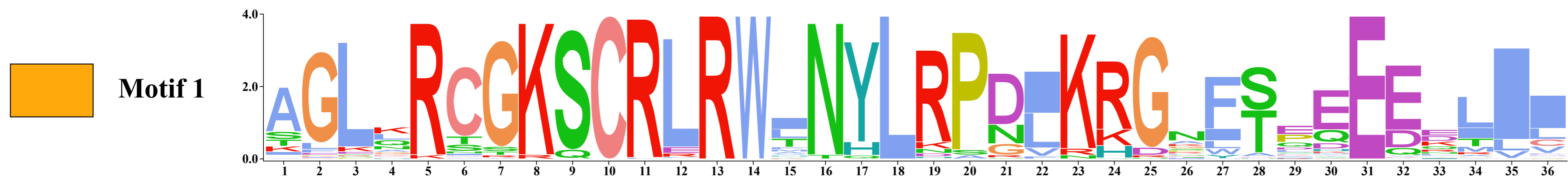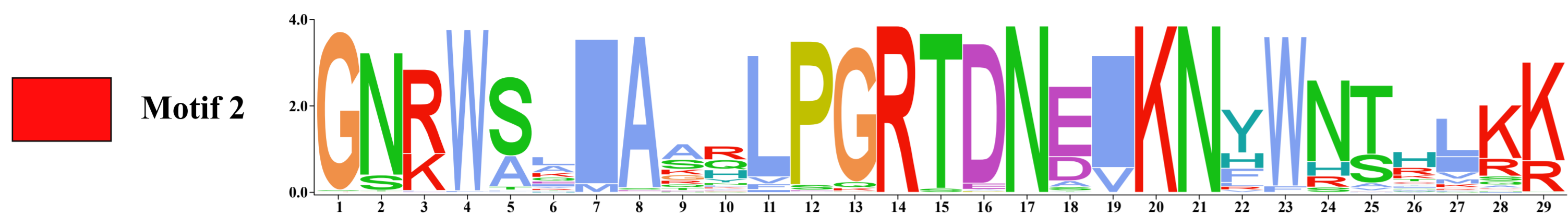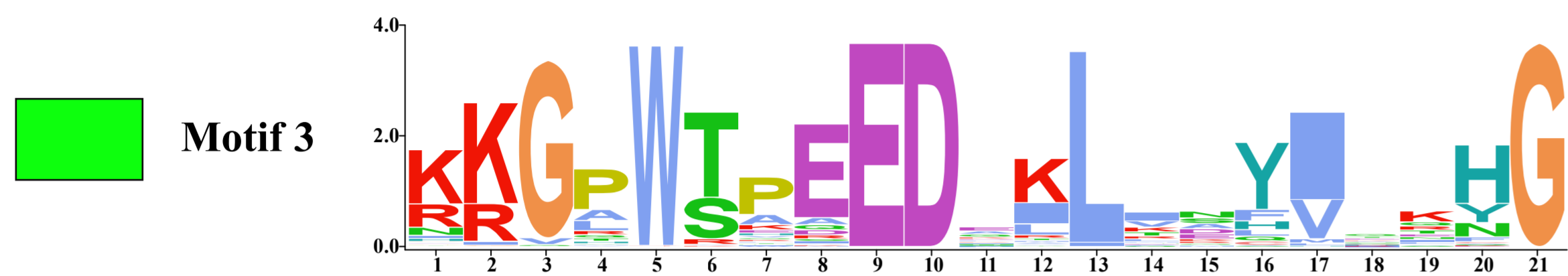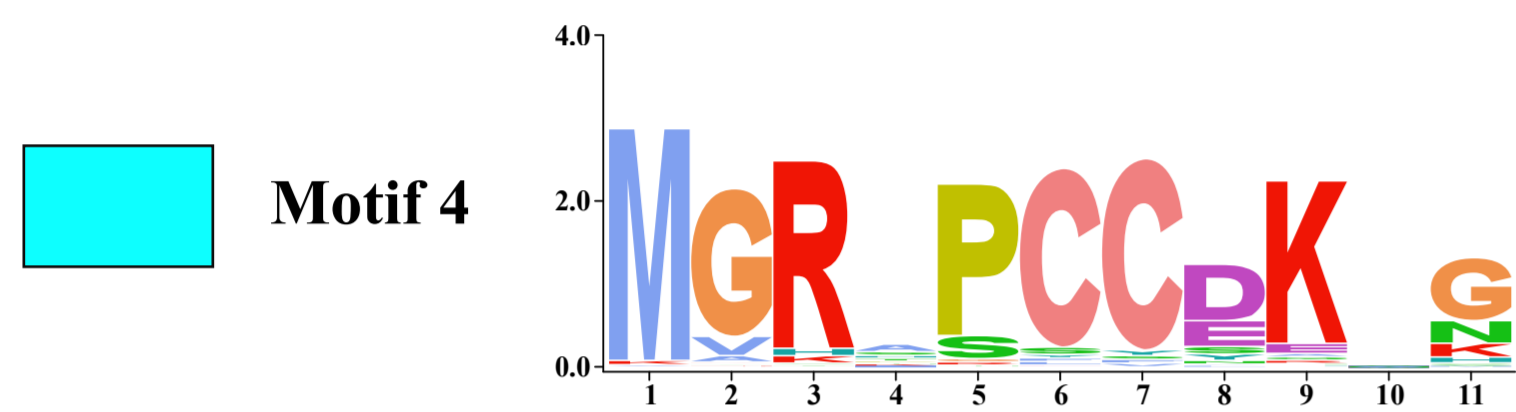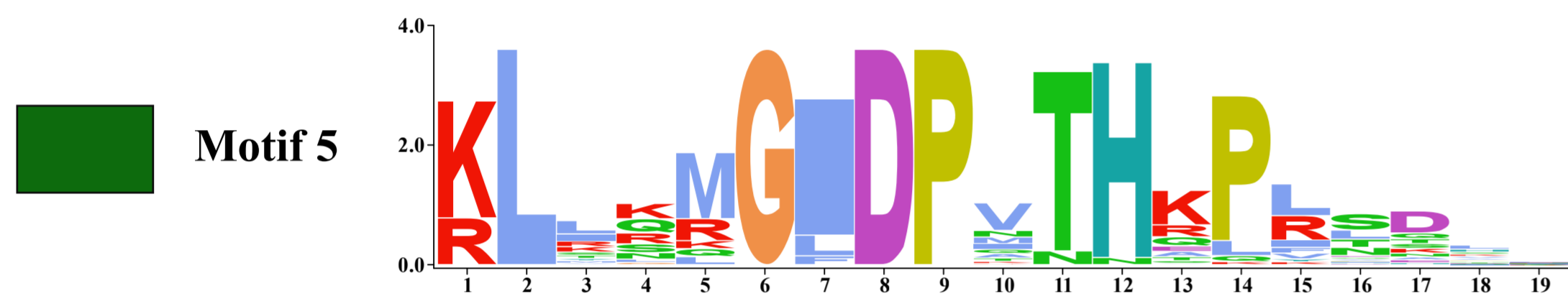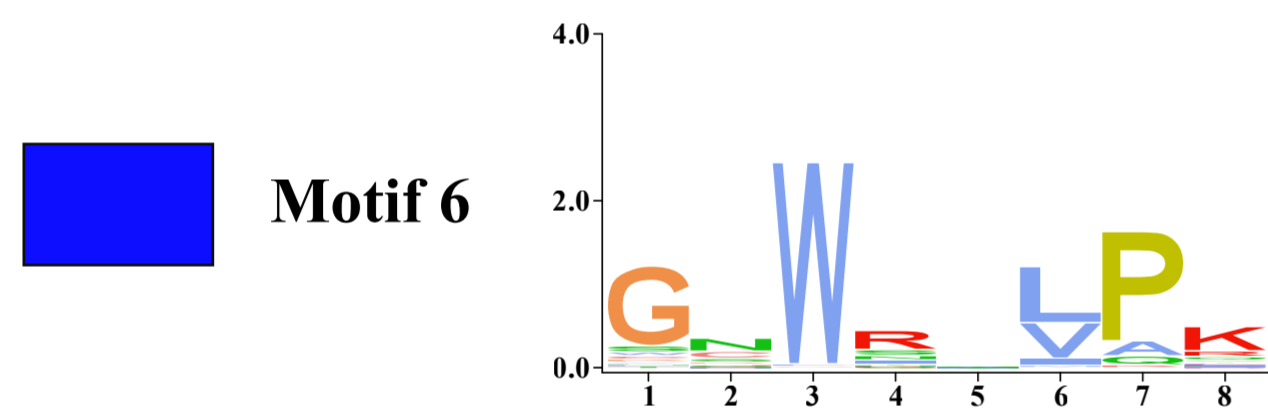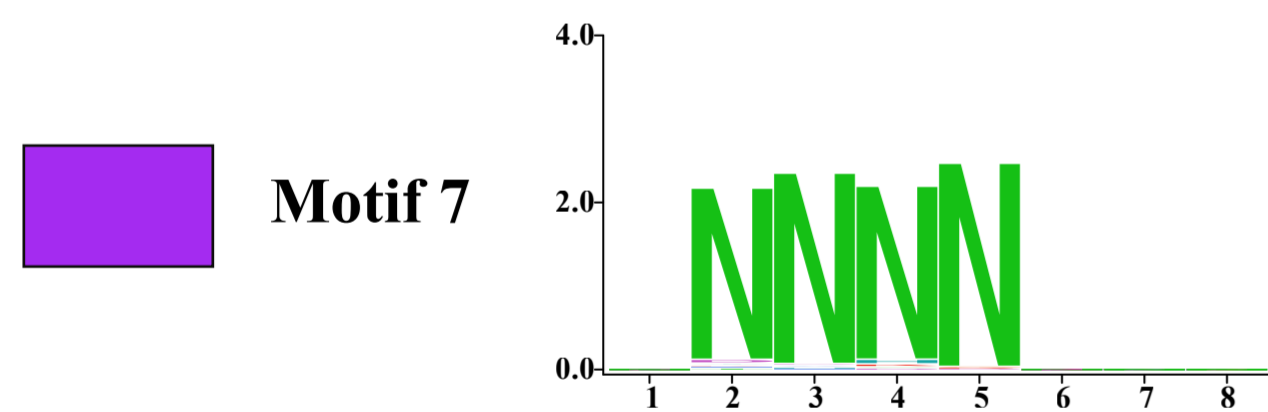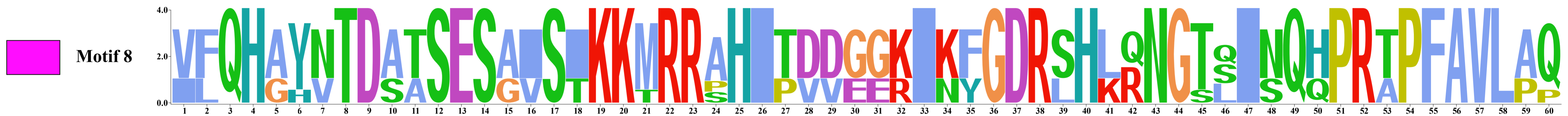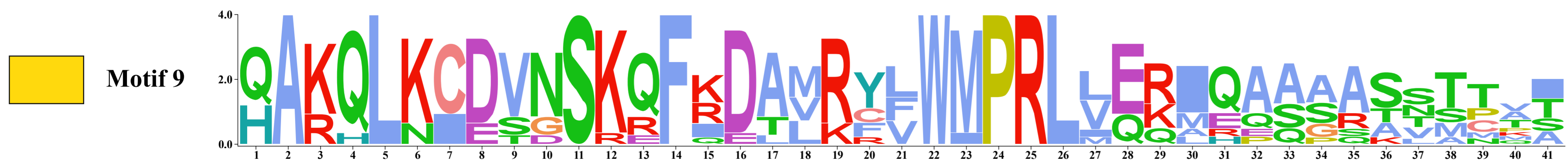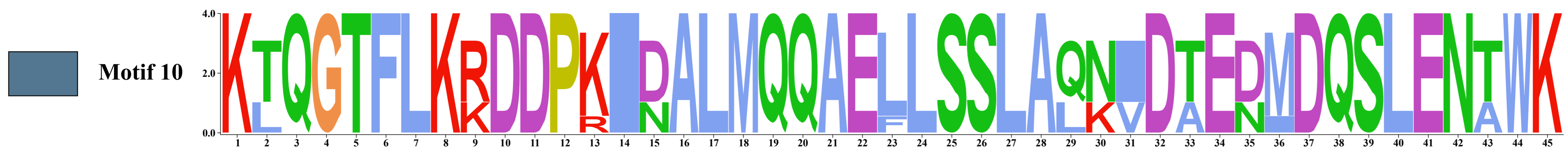

Supplement: Supplementary Figure 5 — 10 MEME motif sequence logos in AhR2R3-MYBs. [file DataSheet_5.pdf]

RNA-Seq qRT-PCR

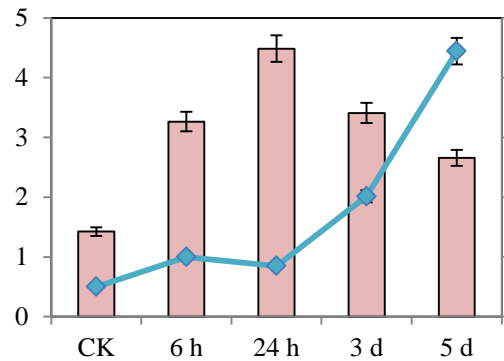

*AhMYB008*

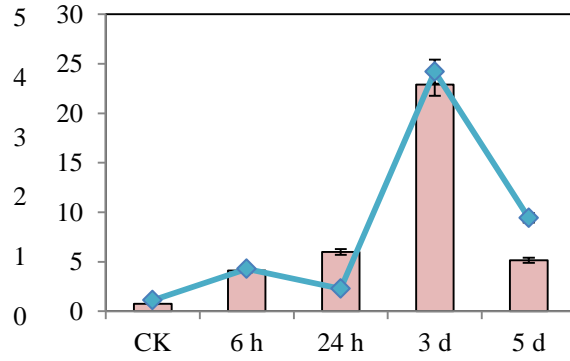

*AhMYB009*

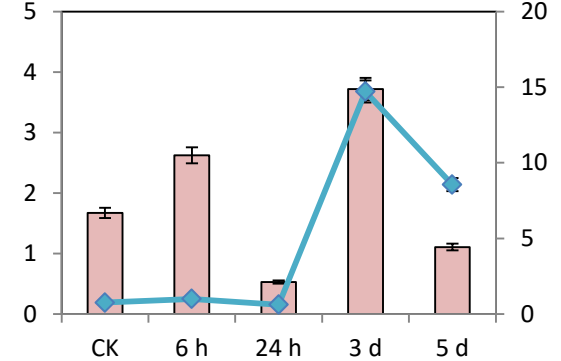

*AhMYB013*

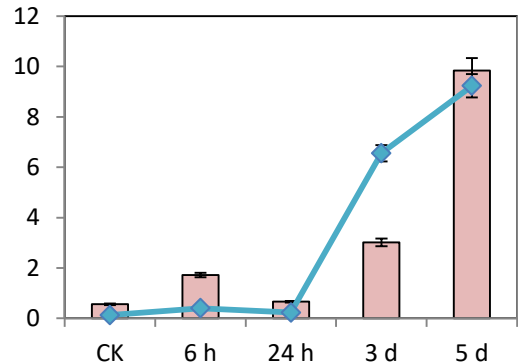

*AhMYB025*

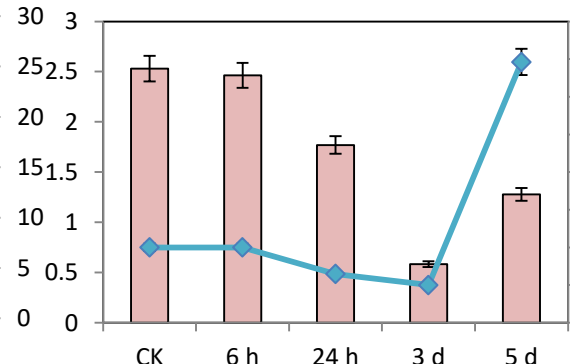

*AhMYB063*

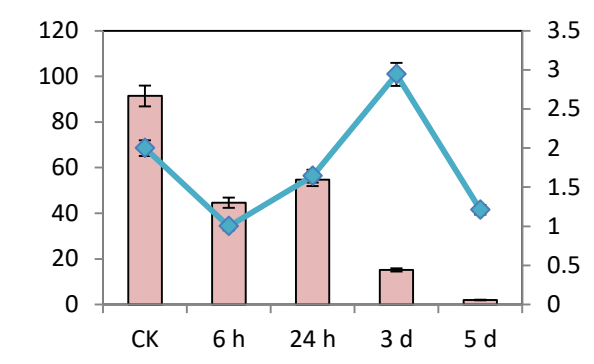

*AhMYB070*

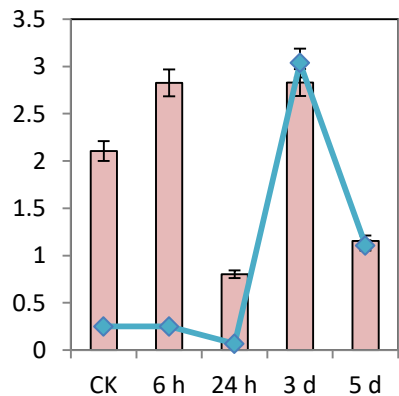

*AhMYB112*

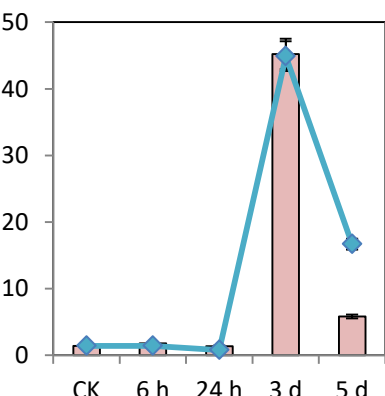

*AhMYB158*

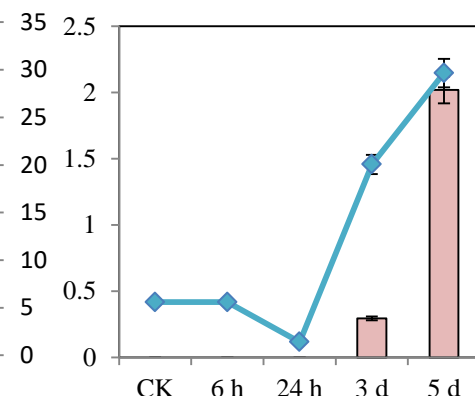

*AhMYB175*

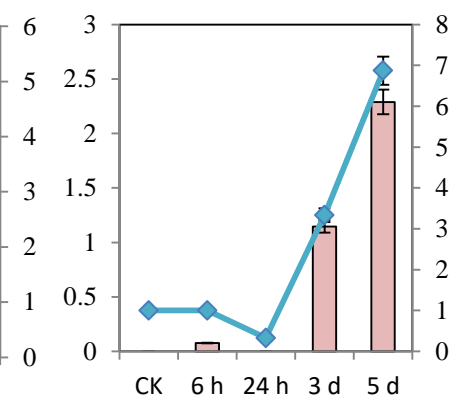

*AhMYB176*

Supplement: Supplementary Figure 6 — qRT-PCR verification of the expression of AhR2R3-MYBs after water logging stress. [file DataSheet_6.pdf]
