# Supplementary material for: Genome-wide analysis of R2R3-MYB genes in cultivated peanut (Arachis hypogaea L.): Gene duplications, functional conservation, and diversification
Source: Front Plant Sci. 2023 Feb 14;14:1102174. doi: 10.3389/fpls.2023.1102174 (PMC9971814; doi:10.3389/fpls.2023.1102174)

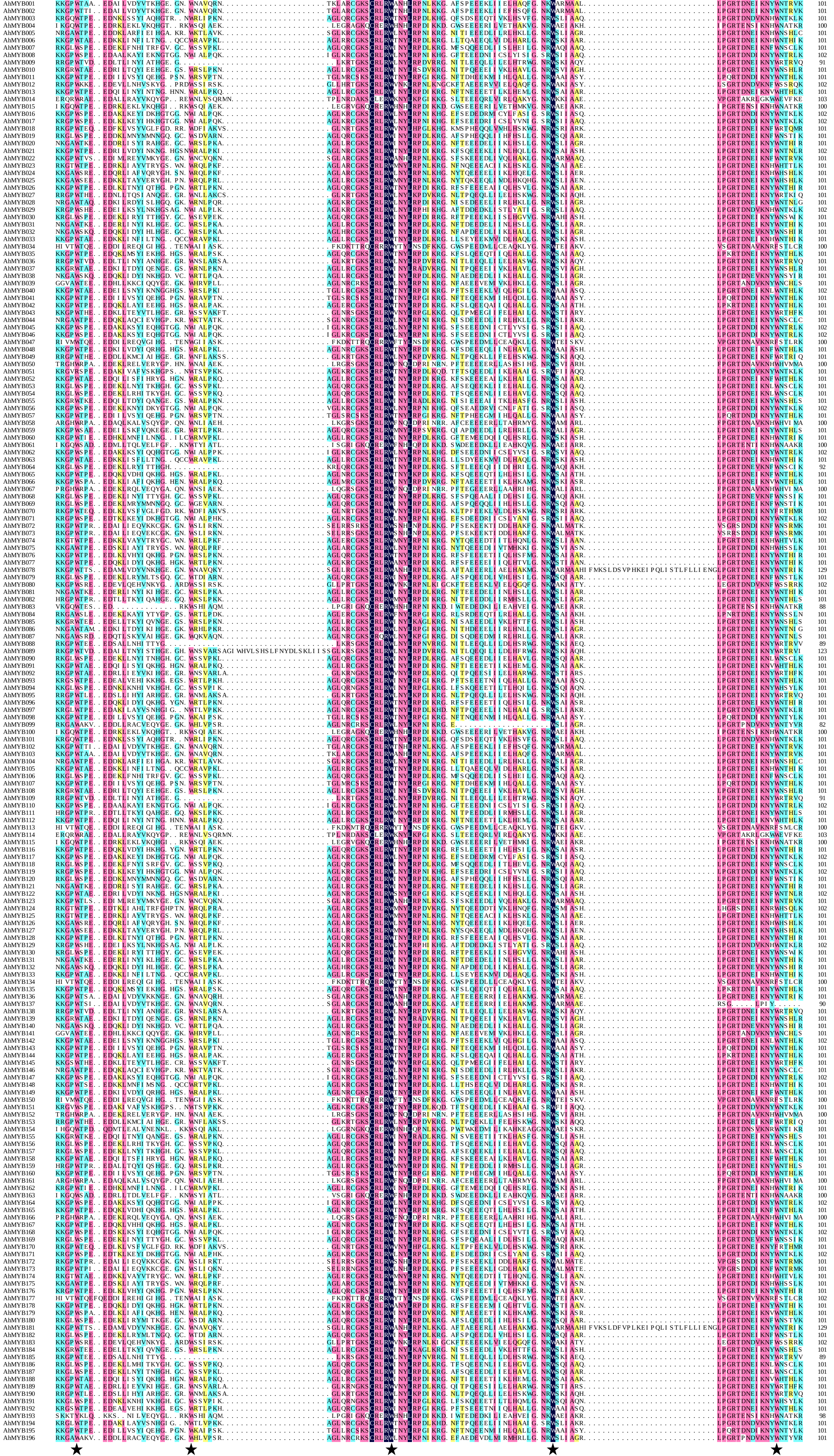

Supplement: Supplementary Figure 1 — Consistent sequence alignment of the R2R3-MYB domain in Arachis hypogaea. The alignment of 196 AhR2R3-MYB domains was performed using ClustalX and DNAMAN. The shading of the alignment represents different degrees of conservation among sequences, respectively. The sites of five highly conserved tryptophan residues (W) were indicated by pentacles. [file DataSheet_1.pdf]
